# Stereo‐cell: Advancing spatial single‐cell biology towards clinical translation

**DOI:** 10.1002/ctm2.70480

**Published:** 2025-09-23

**Authors:** Christian Baumgartner

**Affiliations:** ^1^ Department of Computer Science and Biomedical Engineering Institute of Health Care Engineering with European Testing Center of Medical Devices Graz University of Technology Graz Austria

## Abstract

Stereo‐cell is a newly developed platform for spatial single‐cell sequencing that integrates morphology, transcriptomics, and proteomics in high resolution. By preserving spatial context while enabling multimodal profiling, it bridges the gap between advanced omics and traditional pathology, supporting the detection of rare cells and clinically interpretable diagnoses. This letter highlights the technical innovations of Stereo‐cell, its positioning within the spatial omics landscape, and its potential for precision medicine. Important challenges in data integration, regulatory compliance, and digital modelling are discussed as essential steps on the path to clinical implementation.

## 1 STEREO‐CELL: A NEW ERA IN SPATIAL SINGLE‐CELL BIOLOGY?

The recent publication of *Stereo‐cell: Spatial enhanced‐resolution single‐cell sequencing with high‐density DNA nanoball–patterned arrays* in *Science* by Liao and colleagues marks an important milestone in spatial single‐cell biology.^1^ Building on high‐density DNA nanoball (DNB)–patterned arrays, Stereo‐cell captures intact cells on the chip, records their morphology through imaging, and anchors molecular signals in situ, enabling spatially resolved multimodal analysis. By integrating morphological imaging with transcriptomic and protein profiling, Stereo‐cell operationalises the concept of ‘stereoscopic cells’ proposed by Wang et al.^2^ Unlike traditional single‐cell sequencing methods, which strip cells of their spatial and contextual identity, Stereo‐cell preserves spatial and morphological context while delivering molecular depth and scalability. This restores interpretability, which is central to pathology, and expands the application scope of modern multi‐omics.

## 2 TECHNICAL IMPLEMENTATION AND VALIDATION

Stereo‐cell uses DNA nanoballs (DNBs) with a diameter of ∼220 nm, arranged at intervals of ∼500 nm on a chip surface, where mRNAs released from lysed cells hybridise with DNB‐bound primers while retaining their spatial barcodes.^1^ This design limits lateral RNA diffusion and supports accurate transcript assignment with near‐subcellular resolution.

In benchmark experiments with peripheral mononuclear blood cells (PBMCs), Stereo‐cell generated gene expression profiles that largely agreed with reference flow cytometry data, while droplet‐based single‐cell RNA sequencing platforms tended to overrepresent certain populations such as monocytes. Using large‐format chips, the system profiled up to ∼445 000 cells in a single run, enabling the detection of rare hematopoietic stem and progenitor cells (∼.05%) consistent with known physiological frequencies.

The Stereo‐cell‐CITE extension integrates antibody‐derived tags (ADTs) to enable simultaneous RNA and protein profiling. Surface markers such as CD13, CD14, CD19, and CD88 matched transcriptomic clusters, while additional activation states (e.g., CD112 T cells, CD103 tissue‐resident subsets) were only distinguishable when protein signals were included.^1^ These results confirm Stereo‐cell as a robust, spatially resolved, multimodal single‐cell platform.

## 3 POSITIONING WITHIN THE SPATIAL OMICS LANDSCAPE

The field of spatial omics has developed rapidly. Sequencing‐based barcoding platforms (e.g., Spatial Transcriptomics (Visium),^3^ Slide‐seq^4^) offer genome‐wide coverage but only limited resolution, while imaging techniques such as MERFISH^5^ and seqFISH+^6^ provide subcellular details but are limited to pre‐selected panels and specialised optics.

Stereo‐cell occupies a hybrid position: sequence‐based and scalable, but also with intact single‐cell resolution, approaching subcellular resolution, while preserving morphology. This alignment with clinical considerations is unique, as it combines molecular complexity with the architecture central to pathology. The approach is consistent with recent calls for translationally oriented spatial omics frameworks.^7^, ^8^

**FIGURE 1:**
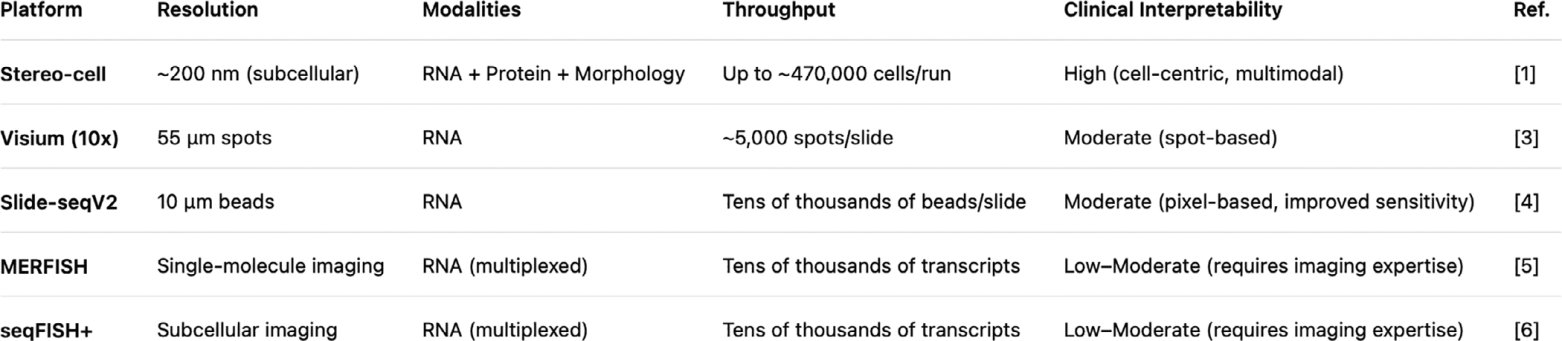
Comparison of spatial omics platforms. Stereo‐cell compared with Visium (10× Genomics), Slide‐seq (version 2), MERFISH, and seqFISH+ in terms of resolution, modality, throughput, and interpretability.

Figure 1 compares Stereo‐cell with Visium, Slide‐seq, MERFISH, and seqFISH+ in terms of resolution, modality, throughput, and interpretability.

## 4 ADVANTAGES OF STEREO‐CELL AND ITS ROLE IN PERSONALISED MEDICINE

Stereo‐cell offers unique advantages that distinguish it from existing spatial and single‐cell platforms.^1^ Its cell‐centric interpretability directly links molecular profiles to morphology, bridging the gap between advanced omics and traditional pathology. Unlike droplet‐based workflows, Stereo‐cell can capture oversized, fragile, or atypical cells, including multinucleated myofibers, neurons with elongated neurites, telocytes, and extracellular vesicles, expanding its applicability to diverse biological contexts.^2^ Its scalability, with the ability to profile hundreds of thousands of cells in a single run, enables reliable detection of rare subpopulations, such as hematopoietic progenitor cells, and provides the basis for building population‐level datasets.^1^

These strengths could be transformative in personalised and precision medicine. By preserving morphology alongside molecular depth, Stereo‐cell enables physicians to associate rare or atypical cell types with disease‐specific features. At a translational level, the scale of the platform supports both the identification of clinically relevant minority populations and the generation of reference datasets large enough to situate individual patient samples within a broader population context. Together, these properties form the basis for integrating Stereo‐cell into clinical diagnostics, where patient‐specific profiles can be interpreted against population‐level standards to support tailored therapies and precision medicine strategies.

## 5 CHALLENGES AND THE DIGITAL TRANSFORMATION OF STEREO‐CELL

The multimodal design of Stereo‐cell generates very large datasets, as each experiment combines transcriptome, proteome, and morphological imaging data for hundreds of thousands of cells in a single run. Both the report by Liao et al.^1^ and the editorial by Wang et al.^2^ emphasise that such data volumes and complexity require advanced bioinformatics pipelines for integration, modelling, and interpretation. Artificial intelligence and machine learning are highlighted as essential tools for extracting clinically relevant insights. In addition, systems biology and computer‐aided modelling approaches have been identified as necessary for simulating dynamic cellular processes and disease mechanisms.^9^, ^10^, ^11^, ^12^ Wang et al. specifically describe *clinical artificial intelligent single cells (caiSCs)* and digital twin models as promising strategies for translating Stereo‐cell results into reproducible and clinically interpretable frameworks.^2^ These challenges underscore the need for scalable computational pipelines and sophisticated modelling frameworks to realise the full potential of Stereo‐cell.

Beyond the current status of the Stereo‐cell approach, clinical implementation requires additional steps that are familiar from digital medicine and large‐scale initiatives such as the Human Cell Atlas.^13^ Patient data must be managed in accordance with the FAIR principles for scientific data management^14^ and secured in infrastructures that comply with standards such as ISO/IEC 27001 for cybersecurity and data protection.^15^ This requires not only sufficient computing capacity (such as high‐performance computing clusters or secure cloud platforms), but also robust governance to ensure interoperability, safe storage, and protected transmission of sensitive health data. As Stereo‐cell data sets are increasingly used in digital twin technologies for predictive modelling and prescriptive simulation,^9^, ^10^, ^11^ their clinical utility depends on rigorous verification, validation, and regulatory oversight. These challenges underscore the need for scalable computing pipelines and sophisticated modelling frameworks to fully leverage Stereo‐cell's multimodal datasets.

## 6 REGULATORY AND STANDARDISATION CONSIDERATIONS

The transition from innovation to clinical application also requires adaptation to existing regulatory procedures. In the European Union, compliance with the In Vitro Diagnostic Medical Devices Regulation (IVDR)^16^ is essential not only for the validation of the physical chip and imaging hardware, but also for demonstrating analytical performance and clinical utility. It is important to note that Stereo‐cell's bioinformatics pipelines and AI‐driven interpretation modules may fall under the scope of ‘Software as a Medical Device’ (SaMD)^17^ and therefore must also be validated in addition to the wet lab components.

In the United States, Stereo‐cell would be treated as an IVD under the Federal Food, Drug, and Cosmetic Act. In addition, the FDA would review AI/ML‐driven analyses under its new SaMD frameworks, such as Good Machine Learning Practice (GMLP).^18^ These guidelines emphasise the need to demonstrate analytical validity and clinical evidence while ensuring continuous monitoring during the further development of the system, which is highly relevant for a platform that integrates RNA, proteins, and morphology on a large scale.

In China, Stereo‐cell would fall under the jurisdiction of the National Medical Products Administration (NMPA), which requires rigorous pre‐market evaluation and post‐market surveillance.^19^ While the NMPA already regulates IVDs, the inclusion of Stereo‐cell's AI‐driven bioinformatics in the diagnostic workflow means that both the hardware and computational components would be subject to scrutiny. The increasing alignment of NMPA processes with international standards offers Stereo‐cell the opportunity to achieve consistent evaluation across different jurisdictions.

Finally, global initiatives led by the International Medical Device Regulators Forum (IMDRF) and the World Health Organization (WHO) are driving the harmonisation of requirements for IVDs and SaMD. Such harmonisation is particularly important for Stereo‐cell, as the company's value lies in generating complex multimodal data that must be validated at both the laboratory and computer levels. Ultimate clinical adoption will depend on how these national paths are pursued and how global standards are maintained to ensure that a stereoscopic, multimodal diagnostic platform is trusted and accepted worldwide.

## 7 A WORLDWIDE INITIATIVE

The transformative power of Stereo‐cell requires coordinated global action. Its introduction should be driven as a worldwide initiative that brings together clinicians, healthcare providers, policymakers, researchers, industry, and patient advocates. Global frameworks will be needed to harmonise regulation, support broad implementation, and ensure worldwide accessibility.

The originators of Stereo‐cell have already initiated an international communication platform – *Stereo‐cell.org* – which is currently under development and will serve as a hub for resources, training, and data exchange to promote transparency and accelerate adoption. Such collaboration will be essential to transform Stereo‐cell from a scientific innovation into a widely accepted diagnostic platform that improves the health of patients worldwide.

**FIGURE 2:**
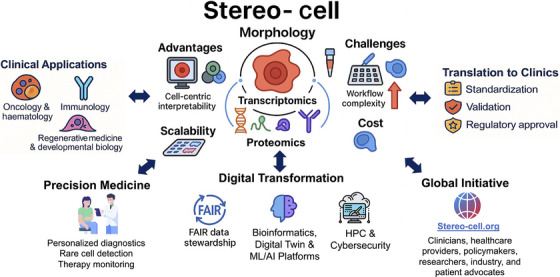
Stereo‐cell: Concept to clinical translation. Stereo‐cell integrates morphology, transcriptomics, and proteomics into a stereoscopic cell record, enabling applications across oncology, immunology, regenerative medicine, and developmental biology. Its strengths include cell‐centric interpretability, inclusivity, and scalability, supporting precision medicine through personalised diagnostics, rare cell detection, and therapy monitoring. Successful clinical translation will require digital infrastructures, including FAIR data stewardship, advanced bioinformatics, AI/ML and digital twin platforms, high‐performance computing (HPC), and robust cybersecurity, together with standardisation, validation, and regulatory approval. A coordinated global initiative (Stereo‐cell.org) involving clinicians, researchers, policymakers, industry, and patient advocates will be essential for worldwide adoption.

Figure 2 illustrates the Stereo‐Cell concept toward clinical translation.

## 8 CONCLUSION

The report by Liao et al. in *Science* validates Stereo‐cell as a multimodal platform and advances the concept of ‘stereoscopic cells’ from vision to practice. By combining morphology, transcriptomics, and proteomics in spatial context, Stereo‐cell offers capabilities beyond existing spatial technologies and delivers clinically interpretable data at scale. With continued progress in digital infrastructure, regulatory alignment, and global collaboration, Stereo‐cell could establish precision medicine in the stereoscopic cell as a new fundamental unit of diagnosis and therapy.

## AUTHOR CONTRIBUTIONS

CB conceptualised and wrote the letter.

## CONFLICT OF INTEREST STATEMENT

The author declares no conflicts of interest.
